# Editorial: Insights in human and medical genomics 2024

**DOI:** 10.3389/fgene.2026.1803517

**Published:** 2026-03-04

**Authors:** Jared C. Roach, Maxim B. Freidin

**Affiliations:** 1 Institute for Systems Biology, Seattle, WA, United States; 2 King’s College London, London, United Kingdom

**Keywords:** epistemology, genomics, knowledge graph, pangenome, personalized medicine, systems biology, translational medicine, variant annotation

**FIGURE 1:**
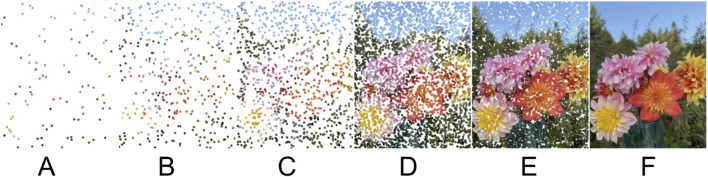
Increasing data density for a systems biology project not only alters quantitative statistical parameters (such as ‘N’—the number of human subjects or possibly samples or data points), but fundamentally and qualitatively transforms the epistemology. Low data density analyses (e.g., **(A)**) rely on assumptions that data points are largely independent and answer queries such as whether bluish points or reddish points dominate. Higher data density (e.g., **(C)**) enables analyses such as machine learning and principal components analysis, with discoveries such as a color gradient along the vertical principal component (PC1), or a pinkish “hub” near the center of the image. Using hypothesis-driven methodology rooted in pre-existing knowledge, assertions such as “presence of sky” may be made, particularly if validated with further research (e.g., **(B–D)**). Human genomics is poised to enter an epoch enabled by very high data density (e.g., **(E)**), revealing holistic mechanistic insight and enabling personalized medicine, but requiring a combination of novel and known analytical methods to reach these insights. Most current projects use data densities more analogous to **(A–C)** than to **(D,E)**.

The field of human and medical genomics is undergoing two major transformations (Figure 1). First, data density and dimensionality is increasing. Second, a combination of new and old analytical techniques—notably empowered by artificial intelligence (AI)—are enabling extraction of mechanistic insight and knowledge from these data. In this editorial we highlight a fraction of the many key papers recently published in this field, with a particular emphasis on those that were invited to our Research Topic with speculative insights based on literature review or research that reflected state-of-the-art methods, detailed recent developments, or highlighted pivotal accomplishments. A focus of this Research Topic was to showcase progress and pinpoint hurdles that need to be surmounted to push the boundaries of what is medically and technically feasible in genetics.

Two years ago, at the launch of this Research Topic, we envisioned potential advances in the following categories: 1. novel genomic technologies and their clinical application; 2. genetic determinants of complex diseases; 3. ethical, legal, and social implications of genomic research; 4. personalized medicine and genomic tailoring; 5. advances in genomic sequencing techniques; and 6. integration of genomic data with AI and machine learning approaches. These fit into a broader arc of progress in our field. Human genomics fundamentally starts with the genome and ends with personalized medicine. Variant annotation remains the foundation of human genomics and must be built on careful observation of patients (Osler, 1912) reported on the scale of case reports (Borovikov et al., 2025) all the way up to population compilations (Ramirez et al., 2022; Sudlow et al., 2015). AlphaMissense provides an example of advancing sophistication in variant annotation (Kurtovic-Kozaric et al.). The insight is that variant annotation cannot end without consideration of complex genomic context. To advance mechanistic insights the field will increasingly incorporate epistasis (Tang et al., 2026), synergies, population and ancestry context (Freidin and Roach, 2025), and whole genome interactions into this functional foundation. Viewing the genome as a web of interactions, and not as a linear construct is a necessary start. Pangenome representations (Nyaga et al.) illustrate an important step in this direction. Furthermore, The Peruvian Genome Project (Guio et al.) highlights the importance of worldwide pan-population data generation coupled to FAIR data governance (https://www.go-fair.org/fair-principles/). Once a foundation of data is generated, it must be analyzed. The insight is that this analysis must be increasingly mechanistic and holistic, focusing on systems rather than single variables. Generative AI (Changalidis et al.) will clearly be key to such analyses in the near future. Coupling newly generated data to old data is key to epistemology (Roach and Freidin, 2023). Knowledge graphs are currently a valuable approach to curating and storing large interconnected datasets (e.g., Goetz et al.) (Fecho et al., 2025). Once knowledge is generated, it can be used to predict health outcomes, enabling prognosis and guiding diagnosis and treatment (e.g., Wu et al., Xiao et al., and Hong et al.). The insight is that machine learning and AI will increasingly drive diagnosis, and that tools must provide explainable mechanisms to maximize their utility. These will lead to transformation in translational medicine and clinical care (Keels et al.; Kroon et al.). The insight is that the evidence-based medicine paradigm of the late 20th century (Sackett et al., 1996) will now link in with the personalized medicine paradigm of the early 21st century (Osler, 1912), to complete one cycle of paradigms and begin another.

Self-critique of the field must address major questions:

*Are current omics data collections big enough?* Dense data drives 21st century research (Roach et al., 2022). Even though modern data sets may comprise data on thousands of variables (e.g., a combination of plasma proteomics and metabolomics), these may not be enough to truly illuminate the dynamic state of the entire human system and its environment. A combination of increasing technological efficiency, brute force of scale, and careful hypothesis-driven selection of assayed biological targets will be necessary to drive complete insights (e.g., shifting from left to right in Figure 1).

*Are we examining enough dimensions?* Many analyses focus on genomics coupled to blood proteomics, metabolomics, and epigenetics. The microbiome is gaining increasing attention. For better systems understanding, we will need to look at multiple tissues and cells (and their compartments) simultaneously, and to measure the entire system dynamically over time. Larger, less biased, recruitment must occur in clinical trials and studies. Continuous monitoring devices and solutions that enable remote trials with massive population participation will drive the future.

*Are we integrating data to gain knowledge and understanding?* As analyses get increasingly complex, it becomes more important to carefully design controls, and to red-team (systematically testing for flaws or biases) the computational workflows. Control analyses limit the ways our complex workflows can fool us into conclusions not supported by the data. The safeguards provided by controls become critically important given the widespread availability of AI- and ML-tools that automate data handling without necessarily exercising judgment or understanding. We must use systems thinking and rational epistemological approaches such as Hill’s criteria to arrive at medically useful translatable knowledge (Roach and Freidin, 2023).

*Are we creating papers and disseminating data that will be part of large meta-analyses?* As research becomes increasingly collaborative and cross-discipline, we must write manuscripts that will be accessible and understood by lay people, students, experts, and AI agents. Datasets must be publicly available, easing any barriers to high-throughput automated access, following the FAIR doctrine. We must understand the limits of 20th century statistics and adapt to 21st century epistemological needs (Figure 1).

In summary, the developments explored in our highlighted papers as well as in the broader field have redefined our understanding of genetic factors in health and disease and opened new pathways for therapeutics and diagnostics. We are now launching a new Research Topic, “Insights in human and medical genomics 2026” and will revisit our assessment of the stage of the field at the conclusion of this new topic. We invite all to participate and look forward to being dazzled by predicted advances and astounded by the unexpected.
